# Book Reviews

**Published:** 1799-12

**Authors:** 


### POPULAR MEDICINE.

*Lectures on Diet and Regimen, &c.* by Dr. Willich.

[ Concluded from our laſt Number, pp. 393 — 396. ]

"Nature reſents every outrage committed on her treaſures, and ſeldom fails to puniſh the tranſgreſſors with lingering diſeaſe, or early diſſolution. This obſervation may be applied to the moral as well as the phyſical faculties of man. It is commonly ſaid, and not without ſome degree of truth, that very forward children ſeldom live to any age; and that too early an exertion of mental powers is in moſt caſes deſtructive. The ſame remark holds good in what relates to the body. The inhabitants of hot climates, who frequently marry at the age of ten and twelve, or twelve and fourteen, begin to be old at thirty, and rarely ſurvive the ſixtieth year. Every thing which haſtens the evolution of the natural powers, every exertion of ſtrength diſproportionate to the ability of the individual, ſhould be carefully avoided, as of a dangerous tendency. Hence the great art of education, the great art of living, conſiſts in following the path of Nature.

*Fifthly,* We ſhould conſtantly inure ourſelves to the habits of ſupporting and reſiſting the various impreſſions of external agency. Some perſons who have paid a very rigid attention to diet, have, notwithſtanding, been unable to reach even a middling age; while others, who have been addicted to the moſt irregular and extravagant courſes, have been obſerved to live to one very advanced. Hence ariſe contradictory maxims in dietetics, which can only be reconciled by deciding chemically between the two extremes, and aſcertaining pretty nearly the abſolute and relative ſalubrity of things. All deviations from the rules of diet are in a certain degree hurtful; although theſe may, in moſt caſes, have only a limited value. Many epicures have been known to reach thei their ſeventieth and eightieth year, if they have once ſurvived a certain critical period of their lives. As ſoon as the body becomes accuſtomed to the uſe of certain things, at firſt diſagreeable and perhaps hurtful, the noxious tendency will not only be removed, but we ſhall find our frame hardened and ſtrengthened by the habit of uſing them. Nature muſt ſtand many a ſhock, if ſhe would familiarize herſelf to the viciſſitudes of climate and oppoſite modes of life, but every victory ſhe gains in theſe encounters, will be a means of rendering her more vigorous and unconquerable. How could the ſublime mind of FREDERIC the GREAT have remained ſo long in its earthly vehicle, if he had not improved, by conſtant culture and diſcipline, his original diſpoſition to a long life. A thouſand other men, who have endured as much exerciſe of body and exertion of mind in their younger years, have yet not attained to any remarkable age. Severe and obſtinate diſeaſes have alſo been thought, in many inſtances, to contribute to the prolongation of life: this is at beſt, however, but a doubtful point; although it cannot be denied, that many ſick perſons have, to all appearance, acquired additional ſtrength and ſpirits, after having recovered from a diſtreſſing quartan ague, or ſome threatening pulmonary diſorder.

"*Sixthly*, We may take notice of a certain *ſteady and equal progreſs through life*, as highly conducive to the great object in view, whether it flows in the manner of a gentle ſtream, or reſembles the more active courſe of a rapid river. The mind, when accuſtomed to certain ſituations and purſuits, which almoſt conſtantly affect it in an uniform manner, is moſt likely to preſerve its reaſoning powers unimpaired and ſtrong. He whom neither violent joy convulſes, nor deep melancholy corrodes, whoſe drama of life is not chequered by too ſudden viciſſitudes, may, with ſome probability, expect a long enjoyment of that life to which he has become ſo habituated. There are many whoſe days quietly glide away, like thoſe of a ſimple ruſtic, in continual ſameneſs: ſuch perſons, it is obſerved, generally live to a great age.

"*Seventhly*, A very neceſſary cauſe of the attainment of an advanced age is a ſound ſtate of digeſtion. In very old perſons, we generally find the digeſtive organs in excellent condition; nor is there a ſurer ſymptom of approaching diſſolution, than complaints in the ſtomach, or frequent returns of indigeſtion. The Swiſs are indebted, it is thought, to the vigorous tone of their digeſtive organs, for the long preſervation of their lives, in general, and for the great number of aged perſons among them. Milk and vegetable food ſeem remarkably well adapted to invigorate the ſtomach. To effect the ſame purpoſe, Lord BACON adviſes old people to have recourſe to ſtrengthening baths, fomentations, and ſimilar *external* remedies, which operate upon the abſorbent ſyſtem; at the ſame time, a thin but nouriſhing and moderate diet ſhould be obſerved, in order to ſpare the organs of digeſtion.

"*Eighthly*, and laſtly: We may recommend equanimity, or that ſtate of the mind, when, from the happy nature of its purſuits, it is not diſquieted by too violent exertions. In the literary profeſſions, and particularly among ſuch individuals as are placed in eaſy circumſtances, we diſcover as many inſtances of longevity, as in the more laborious occupations. It was remarked by the ancients, that grammarians and rhetoricians commonly attained a great age. The mind being engaged in ſcientific purſuits, and other objects in which it finds pleaſure, ſuch as converſation converfation on literary and mixed topics, collecting the productions of Nature, a continual feries of mental refearch, diverfifying the purfuits or amufements, yet gradually and conftantly perfevering in exertions toward the attainment of fome principal object,—all fupply the vital power, as it were, with materials, like the crufe of oil, which proved a never-failing fupport to the widow of *Sarepta*. On the other hand, it is a general remark, that deep thinkers, fpeculative philofophers, and thofe whofe powers are continually abforbed in abftrufe inquiry, foon feel the effects of age from the great exertions of their mental powers. This muft be underftood, however, with exceptions, as in the cafes of Sir Isaac Newton, Haller, Euler, and, the pride of his nation and age, the profound and venerable Kant, ftill living at Koenigfburg.

It would, perhaps, be confidered prefumptuous, if we were to give farther extracts from the fubfequent chapters of this work, in which the author treats, at large, of Air and Weather; Cleanlinefs; Drefs; Food, Drink, and Spices; Exercife and Reft; Sleeping and Waking; Evacuations; Sexual Intercourfe; the Paffions and Affections of the Mind; the Organs of Senfe; and, the Treatment and Prefervation of the Eyes. —To the 'Conclusion,' he has fubjoined the following *Corollary:*

"A luxurious life, and diffolute manners, not only impoverifh a people, but ultimately depopulate the country. Such mifchievous confequences can be averted only by laws wifely enacted, duly adminiftered, and experimentally adapted to the natural capacity and difpofition of a people: for, if their artificial propenfities and defires be not controlled in time, and directed to ufeful ends, the citizen muft degenerate into a feeble and irrefolute flave, and his progeny will gradually wither away, like a plant in a foreign foil. Thus Rome was fubdued, when fhe departed from her ancient fimplicity of manners, when fhe adopted foreign and effeminating refinements, and when her feafts and public amufements became too frequent."

In a 'Poftfcript' the author informs the public, that he purpofes, next year, to publifh another volume, intended as a counterpart to thefe Lectures. "Having treated in the prefent volume, (fays he) of almoft every fubject that relates to the management of the human body in its *healthy* ftate, my next work fhall be entirely appropriated to its treatment in a *difeafed* ftate. It fhall comprehend an accurate and clear defcription of difeafes, together with a plan founded on the rules of experience, how to treat and eventually to cure them, efpecially thofe of a chronic nature. The adminiftration of medicine ought, in fuch a work, to be only a *fecondary* mean of removing difeafe; as it will be admitted by the moft enlightened and candid of the profeffion, that, by ftrictly medical remedies, we can cure *fymptoms*, and afford occafional alleviation of pain; but that we cannot affect a favourable change in the nature and progrefs of a difeafe, without due attention to food, drink, air, fleep, exercife, or reft, &c.

*A few*

*A few practical Remarks on the Medicinal Effects of Wine and Spirits; with Observations on the Oeconomy of Health: intended principally for the Use of Guardians and others intrusted with the care of Youth:* By W. SANDFORD, Surgeon to the Worcester Infirmary. Small 8vo. 152 pp. *London.* Cadell and Davies.

We think this benevolent work well worthy the most serious attention of those for whose use it is written; and even the medical practitioner will have no cause to regret the time he bestows on its perusal.

---

*Bako Von Verulam über die Lebensverlängerung*:—Bacon of Verulam, on the Prolongation of Life. Translated and illustrated with Remarks, by C. A. STRUVE, M. D. 1799. 8vo. 264 pp. (16 gr. or 2s. 8d.) *Glogau.* Günther.

The illustrious Bacon, who opened new paths of inquiry in various branches of science, also taught his cotemporaries how to promote the beneficial application of physical and historical science, and extend their influence on the happiness of mankind. He not only wrote a profound and valuable work on this subject, entitled, "*Historia vitæ et mortis*," but he also evinced, by his own example, under what circumstances and conditions, man may attain the greatest possible age, and how he may secure his health, together with that of his numerous posterity, against the inroads of disease. He demonstrated, that speculations unsupported by facts, must necessarily mislead us in our investigations of physical objects. We cannot but admire the truly philosophic spirit that prevails throughout his numerous works, as well as in that which has given birth to the present publication; for it is not strictly a translation, but a learned commentary of Bacon's "Historia vitæ et mortis," which Dr. Struve has occasionally abridged, enlarged, and illustrated with remarks conformable to the spirit of the age, and consistent with the present state of Medical Science. The German Commentator was of opinion, that his work would serve as a proper counterpart to Professor Hufeland's valuable '*Macrobiotica*, or the Art of Prolonging Life,' a work that has lately been translated into English, while another much improved and enlarged edition of it appeared in Germany.

Every reader who is acquainted with Bacon's acute and correct method of reasoning; his sagacity of discriminating between fact and opinion, truth and fallacy, sophistry and valid argument; his uncommon perspicuity in arranging facts, and deducing from them the most striking and important conclusions; will, doubtless, agree with us, that Dr. Struve deserves the thanks of his cotemporaries, for having undertaken the arduous task of transflating and adapting this judicious treatise to the prevailing taste and opinions; and thus contributing to revive the study of his invaluable works, which, in the present age of hypothetical frivolity, are but too much neglected. It is, however, our duty to remark, that Dr. S. has not bestowed that degree of diligence and industry in translating and illustrating this Latin treatise, which characterises most of his other works on medicine, addressed to unprofessional readers. We mentioned in our first Number, p. 84, that the Royal Humane Society of London had presented this popular writer with a copy of their works, for his excellent treatises on re-animation and and other subjects connected with the philanthropic views of the Society: grateful for this mark of attention shewn to him by so respectable a body of generous and enlightened individuals, Dr. Struve has lately dedicated the following classical work to that illustrious Society:

*Versuch über die Kunst Scheintodte zu beleben, &c.*—An Essay on the Art of re-animating persons apparently dead; and on the means of relief in sudden danger of death. A pocket-book in the form of tables. By Christian August Struve, M. D. 8vo. 150 pp. exclusive of the dedication, preface, and contents. *Hanover.* Hahn.

The modest author informs us in the preface, that he was induced to publish this work, in consequence of the approbation bestowed by the German public on his former writings, relative to this subject. He justly claims the indulgence of the scientific reader, if this attempt should be found deficient in the execution; as he is unfavourably situated with respect to *literary* resources, his place of residence being at a considerable distance from public libraries. His wishes would be much gratified, if the present Essay should excite his countrymen to establish Institutions similar to that of the Royal Humane Society of London, and the Society for the encouragement of useful arts and trades at Hamburgh. He bitterly complains of the want of such Institutions in Germany, while he makes honourable mention of the only Society in imitation of that in London, lately established at Leipzig, by the magistrates of that learned city. Yet he admits that there exist, in Germany, different philanthropic Institutions, such as those for saving persons from fire, in every town of Saxony; the Lying-in Charity at Lübben, in Lower Lausatia, and others.

This compendious work is next addressed to the professional reader, in order to afford him a rational and simple method of treating persons under asphyxia, whether natural or accidental. For this purpose, the tabular form has been adopted by the author as the most proper, especially as it enables the reader, without loss of time, to survey all the rules and cautions, on a sudden emergency.

Those remedies, the application of which is still contested, or which can be resorted to only in particular cases, such as the inspiration of air, venæsection, and tobacco-clysters, Dr. Struve has judiciously classed under a distinct section; as they require the greatest precaution to administer them with success. In this work, he has endeavoured to illustrate chiefly the practical methods of treating persons apparently dead, while it is supposed that medical readers are sufficiently acquainted with the *rationale*, on which this treatment is founded. And in this point of view the author hopes, that his attempt will be received with candour and liberality by the profession. Indeed, there will be little occasion to evince that species of indulgence claimed by the author, *pro captanda benevolentia*; as it would be difficult to find a work which, in the small compass of ten sheets, contains information equally interesting and valuable to the medical practitioner, as well as to the philanthropic assistant.

To enable the reader to survey the multifarious objects treated of in this little volume, we shall here give an outline of its contents.

Introduction: *Section First.* I. Historical account of the Institutions established in various parts of Europe, for recovering the lives of persons apparently dead. II. General ideas on the means of resuscitation.

III. Asphyxia. IV. On fome fpecies of apparent death, occafioned by fudden accidents. V. General principles relative to the fubject of reanimation. VI. Further analyfis of the means of refufcitation. VII. General points to be attended to, in the treatment of accidents, or apparent death. VIII. Circumftances deferving attention in particular cafes of asphyxia.

*Section Second,* Introductory remarks. I. The apparatus for refufcitation. II. Practical principles by which the treatment of the apparently dead, or otherwife unfortunate, is to be regulated. III. General treatment of fuch perfons. IV. Particular directions and rules. V. Tables exhibiting the means employed in the different kinds of afphyxia. VII. A fyftematic view of thefe means, according to their effects on the human body. VIII. Remarks on refufcitation, illuftrated with fuccefsful cafes.

*Section Third,* On the means to be employed in fudden and dangerous accidents. I. General and particular fymptoms and circumftances to be inquired into, refpecting the nature of fuch cafes; as, hydrophobia, the different fpecies of poifon and apoplexy. II. Tables exhibiting the means to be reforted to, according to the nature of the cafe. III. Remarks on the prevention of hydrophobia.—An alphabetical lift of the moft remarkable poifonous plants.—A cafe of a perfon poifoned by arfenic, &c. Laftly, Retrofpect of the different remedies fuggefted in this work, and how far they may be employed with fafety, viz. 1. Venæfection. 2. Opening of the trachea. 3. Clyfters. 4. Fluids introduced into the ftomach by a fyringe. 5. Baths. 6. Shower-bath. 7. Bed of afhes or fand. 8. Earth-bath. 9. Friction. 10. Electricity. 11. Infpiration of air. 12. The Tincture of the *Scarabæus majalis,* or *Meloë profcarabæus et Meloë majalis of Linnæus.* 13. The root of the belladonna. 14. Mercurial ointment, in hydrophobia. 15. Soap-ley. 16. Water faturated with hepatic air. 17. Hahnemann's *liquor vini probatorius.* 18. Precautionary rules for recovering perfons fuffocated in pits. 19. Purification of the air in damp apartments.

